# P-395. Serum Uric Acid Dynamics in Chinese HIV Patients: A 20-Year Cohort Study

**DOI:** 10.1093/ofid/ofaf695.612

**Published:** 2026-01-11

**Authors:** Xin Huang, Fada Wang, liyuan Zheng, Xiaojing Song, Wei Lv, Wei Cao, Taisheng Li

**Affiliations:** Peking Union Medical College Hospital, Beijing, Beijing, China People's Republic; Peking Union Medical College Hospital, Beijing, Beijing, China People's Republic; Peking Union Medical College Hospital, Beijing, Beijing, China People's Republic; Peking Union Medical College Hospital, Beijing, Beijing, China People's Republic; Peking Union Medical College Hospital, Beijing, Beijing, China People's Republic; Peking Union Medical College Hospital, Beijing, Beijing, China People's Republic; Peking Union Medical College Hospital, Beijing, Beijing, China People's Republic

## Abstract

**Background:**

The impact of HIV infection and antiretroviral therapy (ART) on serum uric acid (UA) levels and related complications remains unclear. This study aims to investigate the incidence and dynamics of hyperuricemia in people with HIV (PWH) and to explore its clinical correlates within a real-world setting in China.

**Figure 1.**
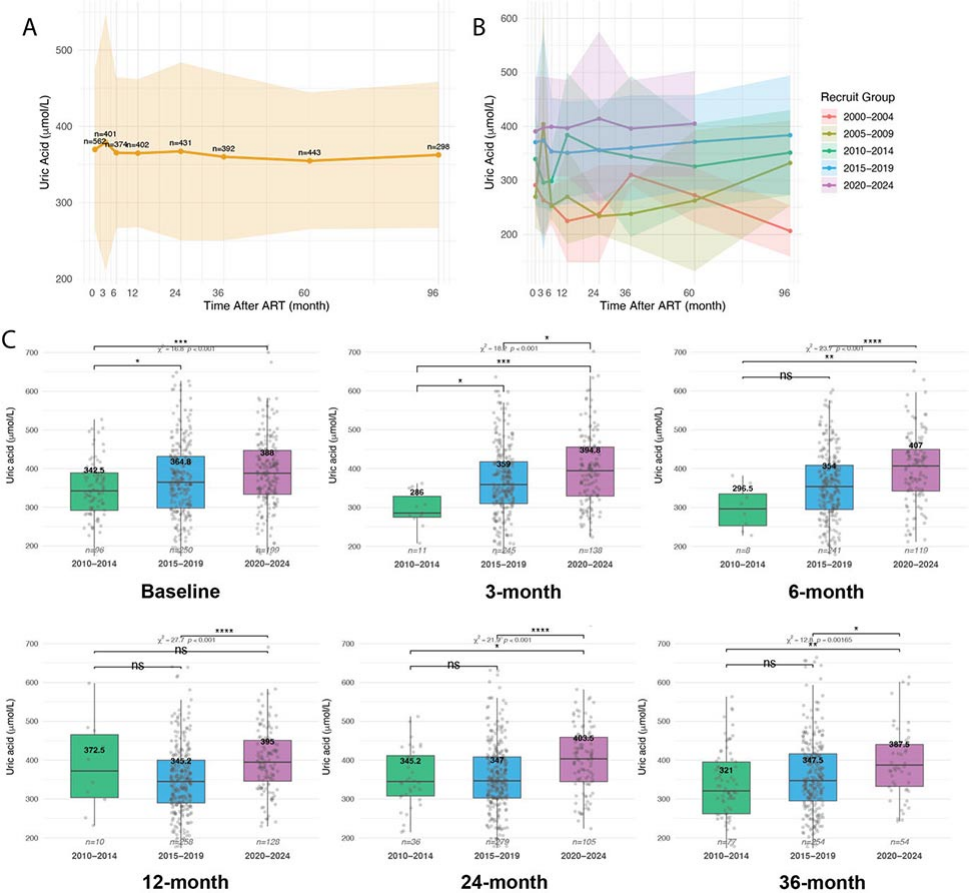
Uric acid (UA) levels over time following treatment in treatment-naïve patients (A) and (B) depict trends in uric acid (UA) levels over time after treatment initiation in treatment-naïve patients. Solid lines represent the mean UA levels (μmol/L) at each time point (0, 3, 6, 12, 24, 36, 60, and 96 months), with shaded areas indicating ±1 standard deviation (SD). Panel (C) shows box plots of UA levels stratified by recruitment period. Significance markers indicate results from post hoc Dunn’s tests with Bonferroni correction for multiple comparisons. Effect sizes (Cliff’s δ) are provided for key pairwise comparisons. Treatment-naïve patients were defined as individuals meeting both of the following criteria: （1） No documented exposure to antiretroviral therapy (ART) prior to baseline enrollment, （2） Initiation of standard ART regimens within the first month post-enrollment.

**Table 1.**
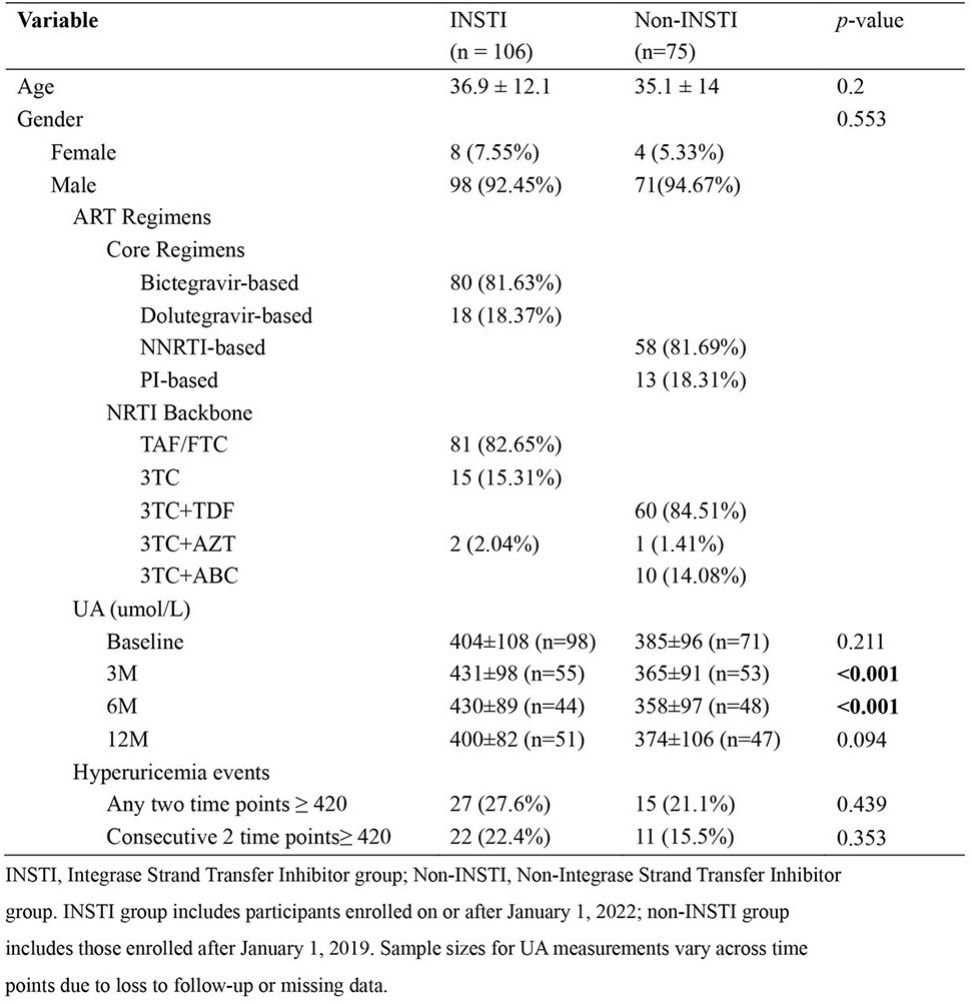
Comparison of Baseline Characteristics and Uric acid (UA) Levels Between INSTI and Non-INSTI Groups in Male Patients

**Methods:**

This study retrospectively analyzed data from a prospective cohort of PWH in Peking Union Medical College Hospital outpatient clinic (2004–2024). A total of 784 PWH were included in the study with complete baseline and follow-up clinical and laboratory data. A Kruskal-Wallis test with Dunn’s post-hoc comparisons revealed group differences, while Cliff’s delta effect sizes quantified magnitude. Generalized Estimating Equations (GEE) with an exchangeable correlation structure were used to assess the change on UA levels, adjusting for baseline UA, age, and sex.

**Results:**

Baseline UA levels differed significantly among PWH enrolled in different eras, with sustained disparities during follow-up. Baseline UA strongly predicted longitudinal changes: each 1-unit increase corresponded to 0.59-unit elevation in follow-up UA (95% CI [0.50–0.68], p< 0.001). Males exhibited 42.6 μmol/L higher UA than females (p< 0.001), while age showed no significant association (p=0.526). Integrase strand transfer inhibitor (INSTI)-based regimens drove UA elevation, with a 39.3 μmol/L increase at 3 months versus non-INSTI groups (95% CI [12.8–65.8], p=0.004). Despite this, hyperuricemia incidence did not differ between INSTI (27.6%) and non-INSTI (21.1%) groups (p=0.439).

**Conclusion:**

UA levels in Chinese PWH have markedly increased in recent years, especially during early treatment. Baseline UA and male gender are key risk factors. Overall health education and targeted monitoring especially with INSTI use in high-risk groups are essential to ensure metabolic safety alongside antiviral efficacy.

**Disclosures:**

All Authors: No reported disclosures

